# Supplementary material for: Lipid Profiles in Preliminary Germinated Brown Rice Beverages Compared to Non-Germinated Brown and White Rice Beverages
Source: Foods. 2022 Jan 14;11(2):220. doi: 10.3390/foods11020220 (PMC8774854; doi:10.3390/foods11020220)
Supplement: Supplementary file 1 [file foods-11-00220-s001.zip › foods-1403251-supplementary.pdf]

foods

Supplemental Table S1. Total lipids (weight %, dwb) and free fatty acids (mg/100g) in three beverages prepared from white, brown and germinated brown 'Rondo' rice, grouped by rice beverage type (GBR, BRR, WR).

Treatments	Total lipid/oil weight % (dwb)	Palmitic & stearic	Oleic	Linoleic	Linolenic	Unknown FFA	Total FFA's
BRR (→ GBR) [1]	*3.95 z* [2]	*29.98 z*	*27.67 z*	*26.64 z*	*1.61 z*	*2.04 z*	*87.93 z*
**GBR (control)**	2.46 yb	24.69 yb	11.83 yb	9.44 yb	0.50 yb	1.33 yb	47.80 yb
PWM	1.98 *B	14.45 *A	5.37 *A	8.81 *A	0.52 *A	0.70 *B	29.85 *A
PNZ	0.62 *t	20.85 *s	6.72 *s	26.03 *t	1.40 *t	0.33 *t	55.33 *s
PWM-SL	5.17 *z	48.08 *Z	16.14 *Z	33.66 *Z	1.98 *Z	3.66 *Y	103.51 *Z
PNZ-SL	n.s. [3]	n.s.	n.s.	n.s.	n.s.	n.s.	n.s.
**BRR (control)**	2.78 a	21.59 b	15.74 b	13.18 b	0.71 b	1.94 a	53.15 b
PWM	2.17 *A	6.85 *B	1.18 *B	1.51 *C	0.16 *B	1.64 A	11.33 *B
PNZ	0.23 *s	5.61 *r	1.91 *r	7.40 *s	0.43 s	0.20 *s	15.55 *r
PWM-SL	3.93 *Y	18.96 Y	3.92 *Y	5.90 *Y	0.42 Y	4.64 *Z	33.84 *Y
PNZ-SL	3.26 *T	66.05 *S	24.05 *S	116.84 *S	5.83 *S	2.37 *T	215.15 *S
**WR (control)**	0.77 c	138.98 a	199.17 a	178.59 a	6.09 a	0.23 c	523.06 a
PWM	0.14 C	5.93 *B	7.09 *A	5.57 *B	0.18 *B	0.08 *C	18.85 *B
PNZ	0.14 *s	31.46 *t	13.87 *t	29.07 *t	1.33 *t	0.22 *t,s	75.95 *t
PWM-SL	i.s.	i.s.	i.s.	i.s.	i.s.	i.s.	i.s.
PNZ-SL	0.94 S	123.49 T	82.50 *T	170.52 T	7.35 T	0.82 *S	384.68 T

[1] Treatment acronyms: BRR, brown 'Rondo' rice; GBR, germinated brown rice; PWM, post wet milling; PNZ, post enzymes; PWM-SL, post wet milling sieving loss; PNZ-SL, post enzymes sieving loss and WR; white rice. Measured factor acronyms: FFA, free fatty acid(s).

[2] Means highlighted with an asterisk (*) are significantly different from the control according to a Dunnett's test at $p<0.05$. Control and treatment means not connected by same letter are significantly different among them according to a Tukey-Kramer HSD test at $p<0.05$. z,y indicates significant differences between germinated GBR control versus the initial BRR used for germination; a,b,c indicates differences among the GBR, BRR and WR controls; A,B,C indicates differences among GBR, BRR and WR for the PWM treatments; X,Y,Z indicates differences among GBR, BRR and WR for the PWM-SL treatments; r,s,t indicates differences among GBR, BRR and WR for the PNZ treatments; R,S,T indicates the differences among GBR, BRR and WR for the PNZ-SL treatment. Data represent means from independent comparisons where n=3 or n=6 ± standard deviation.

[3] n.s. indicates not sampled whereas i.s. indicates insufficient sample to collect.

Supplemental Table S2. Acylglycerols, phytosterol esters (nonpolar lipids), free sterols, oryzanols and summed lipids/oils (mg/100g) in three beverages prepared from white, brown and germinated brown 'Rondo' rice, grouped by rice beverage type (GBR, BRR, WR).

Treatments	TAG (triacylglycerols)	1,3-DAG (diacylglycerols)	1,2-DAG (diacylglycerols)	StE (phytosterol esters)	Free sterols	Oryzanol	Sum of classes (recovered)
BRR (→ GBR) [1]	*3163.52 z* [2]	*24.04 z*	*116.62 z*	*138.50 z*	*35.30 z*	*51.62 z*	*3617.52 z*
**GBR (control)**	1919.15 yb	9.97 yb	52.24 yb	97.29 ya	26.92 ya	25.10 yB	2178.45 yb
PWM	1583.44 *B	15.05* B	64.48 A	81.72 A	24.59 A	18.11 *B	1817.23 *B
PNZ	438.85 *t	9.37 t	7.84 *t	29.02 *t	8.74 *t	5.84 *t	554.98 *t
PWM-SL	3939.34 *Z	19.61 *Y	177.72 *Z	238.55 *Z	50.84 *Z	165.86 *Z	4695.43 *Z
PNZ-SL	n.s. [3]	n.s.	n.s.	n.s.	n.s.	n.s.	n.s.
**BRR (control)**	2356.24 a	23.49 a	80.16 a	102.05 a	22.28 b	35.67 a	2673.07 a
PWM	1844.73 *A	32.03 A	43.49 *B	92.62 A	22.66 A	27.51 A	2074.40 *A
PNZ	171.39 *s	5.39 *s	2.24 *s	20.36 *s	3.43 *s	1.15 *s	219.50 *s
PWM-SL	2999.40 *Y	36.56 *Z	99.90 Y	170.97 *Y	43.62 *Y	132.32*Y	3516.60 *Y
PNZ-SL	2578.79 T	107.36 *T	36.42 *T	137.21 *T	41.91 *T	69.20 *T	3186.03 *T
**WR (control)**	167.89 c	13.39 b	3.40 c	34.42 b	3.35 c	5.66 c	751.17 c
PWM	94.31 C	8.73 C	3.13 C	17.25 *B	2.40 B	1.01 *C	145.67 *C
PNZ	39.59 *s	3.64 s	1.71 s	19.30 *s	2.03 s	0.36 *s	142.55 *s
PWM-SL	i.s.	i.s.	i.s.	i.s.	i.s.	i.s.	i.s.
PNZ-SL	410.12 *S	46.63 *S	16.21 *S	33.42 S	17.23 *S	11.42 *S	919.70 S

[1] Treatment acronyms: BRR, brown 'Rondo' rice; GBR, germinated brown rice; PWM, post wet milling; PNZ, post enzymes; PWM-SL, post wet milling sieving loss; PNZ-SL, post enzymes sieving loss; WR; white rice; CRF, commercial rice flour. Measured factor acronyms: TAG, triacylglycerols; DAG, diacylglycerols; and StE, phytosterol esters including very nonpolar lipids.

[2] Means highlighted with an asterisk (*) are significantly different from the control according to a Dunnett's test at $p<0.05$. Control and treatment means not connected by same letter are significantly different among them according to a Tukey-Kramer HSD test at $p<0.05$. z,y indicates significant differences between germinated GBR control versus the initial BRR used for germination; a,b,c indicates differences among the GBR, BRR and WR controls; A,B,C indicates differences among GBR, BRR and WR for the PWM treatments; X,Y,Z indicates differences among GBR, BRR and WR for the PWM-SL treatments; r,s,t indicates differences among GBR, BRR and WR for the PNZ treatments; R,S,T indicates the differences among GBR, BRR and WR for the PNZ-SL treatment. Data represent means from independent comparisons where n = 3 or n = 6 ± standard deviation.

[3] n.s. indicates not sampled whereas i.s. indicates insufficient sample to collect.

Foods-1403251. John C. Beaulieu, Robert A. Moreau, Michael J. Powell and Javier M. Obando-Ulloa. 2021. Lipid profiles in preliminary germinated brown rice beverages compared to non-germinated brown and white rice beverages.
